# Donor cell-derived genetic abnormalities after sex mismatched allogeneic cell transplantation: a unique challenge of donor cell leukemia

**DOI:** 10.1038/s41408-023-00938-z

**Published:** 2023-11-06

**Authors:** Melanie Klausner, Brian Phan, Laura Morsberger, Rebecca Parish, Alison Shane, Rebecca Park, Christopher D. Gocke, Rena R. Xian, Rick John Jones, Javier Bolaños-Meade, Lukasz P. Gondek, Michael Phan, Ying S. Zou

**Affiliations:** 1 Department of Pathology, Johns Hopkins University School of Medicine, Baltimore, MD USA; 2 The College of William and Mary, Williamsburg, VA USA; 3 Department of Oncology, Johns Hopkins University School of Medicine, Baltimore, MD USA; 4 The Johns Hopkins University, Baltimore, MD USA

**Keywords:** Chromosome abnormality, Cancer genetics

Dear Editor,

Allogeneic hematopoietic cell transplantation (alloHCT) is an important and curative therapeutic approach for patients with hematological malignancies. Given the advancement of alloHCT, disease-free survival after transplant increases dramatically. However, relapse can occur after a transplant. While most relapsed hematological malignancies are of host origin, donor cell-derived leukemia (DCL) emerging from the donor’s hematopoietic stem cells has been infrequently reported. After the first case of DCL was reported in 1971 [1], a low number of cases have been reported in the literature, suggesting it to be a rare phenomenon. The incidence of DCL is ~0.5% (range, 0.08–1) among recipients of alloHCT [2]. DCL has been reported in alloHCT recipients with various hematological malignancies, commonly in acute leukemia [3]. Although there are no exact genetic features for DCL, donor cell-derived genetic abnormalities have been described in DCL patients. Occasionally, acquired/somatic cytogenetic abnormalities found in donor cells after alloHCT have been reported in patients without the development of hematologic malignancies [4, 5], referred to as clonal cytogenetic abnormalities of undetermined significance (CAUS). DCL and CAUS are generally reported as individual case reports, resulting in very few large case series. Acquired genetic abnormalities resulting in DCL and CAUS remain not fully understood given the usual lack of pathological details and follow-ups to distinguish between DCL and CAUS.

We conducted a retrospective study on 2141 patients with hematological malignancies who underwent alloHCT at the Johns Hopkins Hospital for the last 16 years from May 1st, 2006, to December 31st, 2022. This study is focused on genetic abnormalities in DCL and CAUS from 917 patients after sex-mismatch alloHCT (Supplementary Fig. S1). In patients with sex-mismatch alloHCT, karyotype can distinguish between cytogenetic aberrations of the recipient and donor cells (based on opposite sex chromosomes of the recipient and donor). Combinations of karyotype and T cell chimerism/engraftment testing could accurately determine the origin of new leukemia after sex-mismatch alloHCT. Successful engraftment after sex-mismatch alloHCT is defined as pure donor cells by T cell chimerism/engraftment testing, karyotype/FISH for sex chromosomes, and negative pathological reports. We identified genetic abnormalities in donor cells based on karyotype, FISH, chromosomal microarray, T cell chimerism/engraftment, and gene mutation (next-generation sequencing) results [6]. DCL or CAUS status is determined by clinical and pathological data. This study was approved by the Institutional Review Board (IRB) of JHH (IRB#00380308) and performed in accordance with the Declaration of Helsinki.

In this cohort, 38 patients (4.1%) had donor cell-derived cytogenetic abnormalities including 10 (1.1%) patients with acquired cytogenetic abnormalities (Supplementary Table S1) and 28 (3.0%) with germline/constitutional cytogenetic abnormalities. Germline cytogenetic abnormalities included pericentric inversions in chromosomes 2 (inv(2)(p11.2q13), 1 case, 0.1% in this cohort) and 9 (inv(9)(p12q13), 23 cases, 2.5%), Robertsonian translocations (der(13;14)(q10;q10), 2 cases, 0.2%), a supernumerary marker chromosome (+psu idic(15)(q11.2), 1 case, 0.1%), and a balanced reciprocal translocation (1 case, 0.1%). Besides the marker chromosome, all of them have been reported as chromosomal polymorphisms/variants among normal individuals without any clinical phenotype. Depending on the makeup/genomic contents of the marker chromosome, carriers of an isodicentric chromosome 15 could have normal to mild clinical phenotypes such as autism spectrum disorders [7].

Six patients D1–D6 (60% of the patients with donor cell-derived acquired cytogenetic abnormalities, 0.7% of the entire cohort) developed DCL during median of 63.7 months (range 12.4–131.9) after alloHCT, with various cytogenetic abnormalities including monosomy 7/7q deletion (3 patients), gain of 1q (2 patients), t(11;19) translocation involving a *KMT2A* gene rearrangement (Supplementary Fig. S2), marker chromosomes, gain of 21, 22, and 20q deletion (del(20q)) (one patient each) (Fig. 1A and Supplementary Table S2). Three of them had mutations in *ASXL1, CBL, DNMT3A*, *KMT2D*, *IKZF1*, *MPL*, *NF1*, *NRAS, PTPN11, RUNX1, SETBP1*, and/or *TET2* genes in donor cells after alloHCT, with transcription factor *RUNX1* mutations being the most common. D5 had a *NSD1* variant shared in both the patient (before alloHCT) and the donor and a *BCL6* variant found only in the donor cells that has been reported as germline variants in the cancer susceptibility genes of healthy individuals [8]. Five of them died shortly after developing DCL, with median overall survival of 8.3 months (range, 6.9–19.6).

**Fig. 1:**
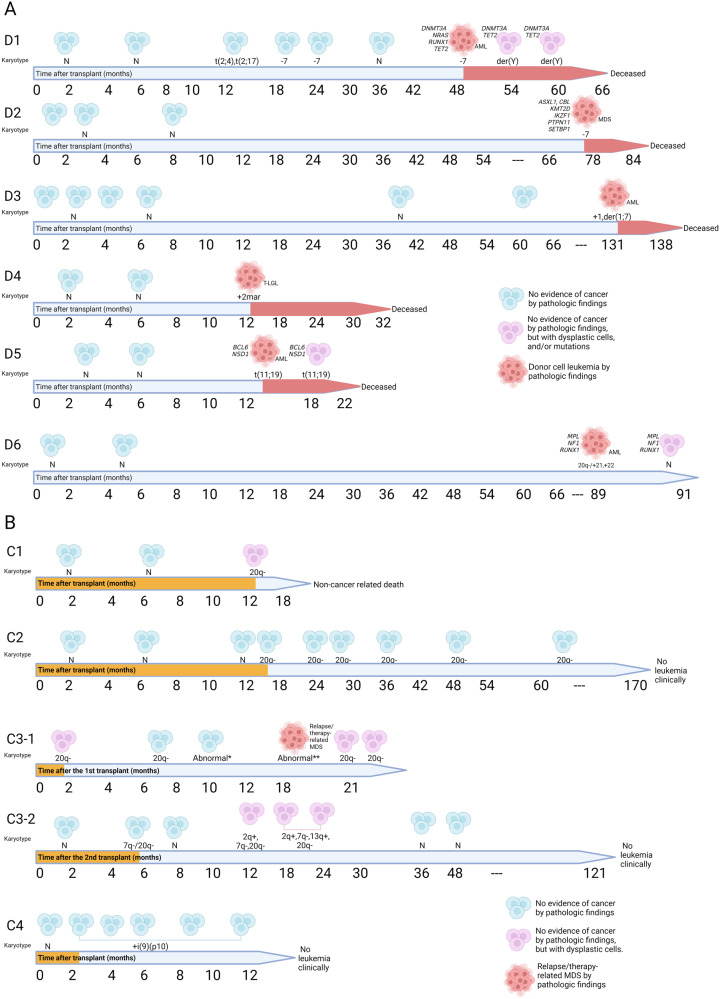
Acquired donor cell-derived genetic abnormalities after allogeneic stem cell transplantation in this study. **A** Acquired cytogenetic abnormalities in patients with donor cell-derived leukemia (D1–D6). **B** Acquired cytogenetic abnormalities as donor cell-derived clonal cytogenetic abnormalities of undetermined significance in C1 to C4 patients. C3 patient had two transplantations and a relapse after the first transplantation.

Four patients C1–C4 (40% of the patients with donor cell-derived acquired cytogenetic abnormalities) had CAUS with no evidence of leukemia and acquired cytogenetic abnormalities including del(20q) (3 donors), gain of isochromosome 9p (resulting in four copies of chromosome 9p, one donor), and a complex karyotype along with del(20q) (1 donor) (Fig. 1B, Supplementary Table S3). Acquired cytogenetic abnormalities occurred shortly after alloHCT (median = 5.7 months, range 1.8–13.3). C3 patient had two alloHCTs using senior donors (mother as the first donor and father as the second donor) and del(20q) observed in both donor cells after alloHCTs. The first donor had del(20q) as the sole cytogenetic abnormality, occurring 1.8 months after alloHCT, and the second donor had del(20q) as a stemline and a sideline along with a 7q deletion, occurring 5.7 months after alloHCT. Clonal evolution of this sideline later showed a more complex karyotype including additional abnormalities on chromosomes 2q and 13q in ~15% of donor cells by FISH within 2 years after alloHCT. With the presence of acquired cytogenetic abnormalities (even in a complex karyotype), pathological findings of bone marrow specimens revealed no evidence of leukemia (occasionally with dysplastic or atypical cells). Two of them had long follow-ups (12 and 14 years, respectively) without leukemia.

Although DCL and CAUS are rare, DCL has a poor prognosis (median survival was <1 year in this study). Various acquired cytogenetic abnormalities were found in this cohort with monosomy 7/del(7q) being common in DCL and del(20q) being frequent in CAUS. Acquired cytogenetic abnormalities occurred earlier in CAUS (median = 5.7 months) than in DCL (median = 63.7 months) after alloHCT (P = 0.001 by the Mann-Whitney-test for independent samples) (Fig. 1 and Supplementary Tables S2, 3). Overlapping cytogenetic abnormalities (such as del(20q) and del(7q) in this cohort) were found in both DCL and CAUS, which makes it very challenging to determine their pathogenesis/clinical significance. We recommend an integrated approach including cytogenetic studies, chimerism analysis, gene mutation testing, flow cytometric immunophenotyping, morphological/pathological findings, and clinical correlation to evaluate evolving DCL (Fig. 2). This combined approach is important to properly interpret the clinical importance of acquired cytogenetic abnormalities after alloHCT.

**Fig. 2:**
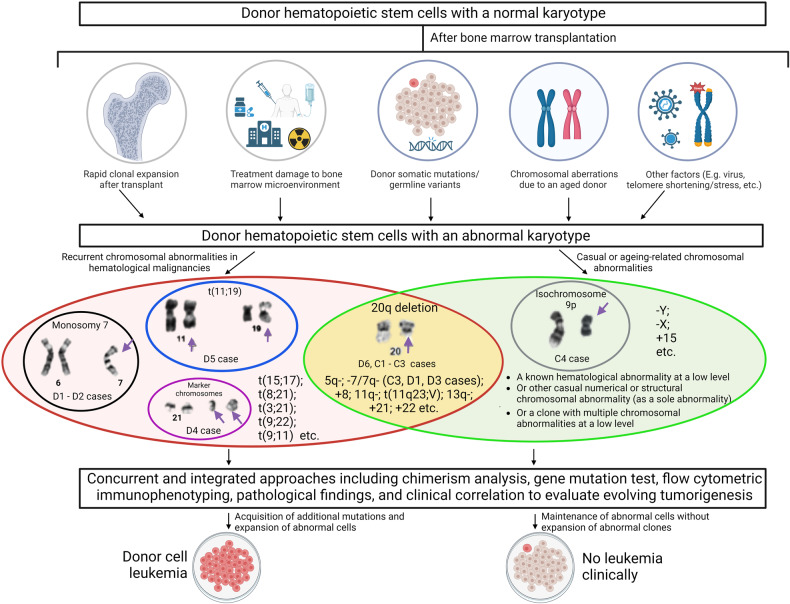
Integrated approaches for understanding pathogenesis of acquired donor cell-derived genetic abnormalities after allogeneic stem cell transplantation. Multifactorial and multiple-hit mechanisms may play roles in development of acquired cytogenetic abnormalities in donor cell-derived leukemia (DCL) and donor cell-derived clonal cytogenetic abnormalities of undetermined significance (CAUS). Although well-known cytogenetic abnormalities associated with hematological malignancies can occur in DCL and CAUS, they tend to appear at a much lower level in CAUS than in DCL. A combined approach including clinical correlation, gene mutations, immunophenotyping, and pathological findings is useful to properly interpret the clinical importance of acquired cytogenetic abnormalities.

The mechanism behind the development of DCL and CAUS is complex and not well understood. Multifactorial and multiple-hit mechanisms have been proposed to link genetic abnormalities in DCL [2, 9], suggesting an interaction among genetic factors (donor and recipient), microenvironment, and transplant procedure triggered leukemic events (Fig. 2). Clonal hematopoiesis of indeterminate potential (CHIP), based on the presence of clones with mutations (variant allele frequency ≥ 2%) in cancer driver genes without overt hematological malignancy, has been described in aging individuals [10]. Donor cell-derived CHIP may be the driver to expand hematopoiesis, and the molecular evolution of donor-derived CHIP clones into DCL has been reported [11]. C3 case had del(20q) clones occurring in two elder donors after alloHCT, suggesting the aged donor’s and/or patient’s microenvironment plays a role in CAUS. While del(20q) is associated with myeloid malignancies, del(20q) has been reported in at least two CAUS cases with follow-ups of 8 to 12 months after alloHCT [4, 5]. Del(20q) is the most frequently detected large structural genetic mosaicism alteration in a healthy population due to advanced age [12]. Although it is not MDS-defining in the absence of morphological dysplasia, it may be associated with age-related clonal hematopoiesis with an increased risk of hematologic malignancy [13]. Levels of reactive oxygen species (ROS) may be affected by aged hematopoietic stem cells, tumor cells, and cellular components, the latter two making up the tumor microenvironment [14]. High levels of ROS may damage DNA [14]. Del(20q) clones in C3 case (two donor cells in the same patient after alloHCT) may be due to aged donor HSCs with pre-genetic abnormality susceptibility potential and may fulfill/accelerate to become a cytogenetic abnormality in the microenvironment of the recipient’s bone marrow.

Donor cell-derived T cell large granular lymphocyte leukemia (DC-T-LGLL) (D4 case) is a rare phenomenon. It is distinguished from reactive conditions based on post-alloHCT chimerism of pure donor cells, a karyotype with donor-sex chromosomes, and a rearranged *TCR* gene [15]. D4 also had acquired cytogenetic abnormalities with a gain of 1 to 2 supernumerary marker chromosomes. Although de novo T-LGLL is usually an indolent neoplasm related to chronic antigenic stimulation from viral infections, GVHD, or immunosuppressants, it has been suggested that DC-T-LGLL may have a different pathogenesis and clinical course [15]. Therefore, it is important to utilize combined pathological and genetic tests to assess emerging DC-T-LGLL in the post-transplant setting.

In conclusion, acquired cytogenetic abnormalities occur ~1.1% in this cohort after alloHCT and occur earlier in CAUS than in DCL. Overlapped cytogenetic abnormalities in both DCL and CAUS hamper determination of their clinical importance. An integrated approach using a combination of genetic, clinical, and pathological data is important to accurately clarify the potential clinical importance of acquired cytogenetic abnormalities in DCL and CAUS.

### Supplementary information

Supplementary Tables (S1–S3)

Supplemental Figure S1

Supplemental Figure S2

## Data Availability

The datasets generated during and/or analyzed during the current study are available from the corresponding author on reasonable request.
