# Supplementary material for: Donor cell-derived genetic abnormalities after sex mismatched allogeneic cell transplantation: a unique challenge of donor cell leukemia
Source: Blood Cancer J. 2023 Nov 6;13(1):163. doi: 10.1038/s41408-023-00938-z (PMC10625970; doi:10.1038/s41408-023-00938-z)
Supplement: Supplementary file 1 — Supplementary Tables (S1–S3) [file 41408_2023_938_MOESM1_ESM.docx]

| **Supplementary Tables (S1-S3)**  **S1: Cases with acquired/somatic donor cell-derived cytogenetic abnormalities** | | | | |  |
| --- | --- | --- | --- | --- | --- |
| **Cases with donor cell-derived leukemia (DCL)** | | | | |  |
| **Case ID** | **Patient disease ^ǂ^** | **Donor information** | **Donor chromosomal abnormalities (karyotype)** | **Donor-derived disease** | |
| D-1 | Non Hodgkin's Lymphoma | brother | //45,XY,-7[19]//46,XY[1] | AML | |
|  |  |  | //46,X,der(Y)t(Y;1)(q11.23;q12)[18] //46,XY[2] |  |  |
| D-2 | Mantle cell lymphoma | brother | //45,XY,-7[16]//46,XY[4] | MDS | |
| D-3 | Aplastic anemia | brother | //46,XY,+1,der(1;7)(q10;p10)[18]//46, XY[2] | AML | |
| D-4 | Multiple myeloma → t-ALL | son | //47~48,XY,+1~2mar[4]//46,XY[16] | T-large granular lymphocyte leukemia | |
| D-5 | AML | sister | //46,XX,t(11;19)(q23;p13.1)[17]  //46,XX[3] | AML | |
| D-6 | CML | sister | //46,XX,del(20)(q11.2q13.2)[7]//46,XX, der(21;22)(q10;q10)x2,+21,+22[11]  //46,XX[2] | AML | |

**^ǂ^** D1-D6 patients had a normal karyotype before alloHCT transplants.

| **Cases with donor cell-derived clonal cytogenetic abnormalities of undetermined significance** | | | | | |
| --- | --- | --- | --- | --- | --- |
| **Case ID** | **Patient disease** | **Patient karyotype/FISH**  **before transplant** | **Donor information** | **Donor chromosomal abnormalities (karyotype)** | **Donor-derived disease** |
| C-1 | MDS | 45,XY,-7[20] and -7 (21%) by MDS FISH | daughter | //46,XX,del(20)(q12q13.3)[5]  //46,XX[15] | No leukemia |
| C-2* | MDS → AML | 46,XX[20] | matched-unrelated | //46,XY,del(20)(q13.1)[10]  //46,XY[10] | No leukemia |
| C-3 | AML (APL) → t-MDS | 45,XX,der(1)t(1;8)(q11;p21),der(8)t(1;8)inv(1(q11q25),-7[20] | mom and father | //46,XY,del(20)(q11.2)[1~11]//46,XY,del(7)(q22q32),del(20)(q11.2)[2]//46,XY, add(2)(q35),del(7)(q22q32),del(20) (q11.2)[2]//46,XY,add(2)(q35),del(7) (q11.2q22),add(13)(q34),del(20)(q11.2) [2~7]//46,XY[8~19] | No leukemia |
| C-4* | ALL | 46,XX[20] | brother | //47,XY,+i(9)(p10)[17~20] //46,XY[0~3] | No leukemia |
| AML: acute myeloid leukemia; ALL: acute lymphoblastic leukemia; CML: chronic myelogenous leukemia; MDS: myelodysplastic syndrome; t-ALL: therapy-related ALL; t-MDS: therapy-related MDS | | | | | |

*: C2 and C4 had myeloablative alloHCT, and remaining eight cases had non-myeloablative alloHCT.

**S2: Acquired/somatic donor cell-derived genetic abnormalities in cases with donor cell leukemia**

| **Case ID** | **Time after trans-plant (days)** | **Cytogenomic data** | **FISH** | **T-cell Chimerism** | **Pathology findings** | **Gene mutations** |
| --- | --- | --- | --- | --- | --- | --- |
| **D-1** | +62 | //46,XY[20] |  | Donor | No evidence of leukemia  (NEL) |  |
|  | +184 | //46,XY[14] |  | Donor |  |  |
|  | +387 | 46,XX,t(2;4)(q11.2;q31.3),t(2;17)(p15;p13)[5]//46,XY[15] |  | Donor |  |  |
|  | +569 | //45,XY,-7[5]//46,XY[18] | XY in 100% | Donor |  |  |
|  | +737 | //45,XY,-7[3]//46,XY[17] | -7 in 12% | Donor |  |  |
|  | +1080 |  | Normal 7 | Donor |  |  |
|  | +1494 | //45,XY,-7[19]//46,XY[1] | -7 in 79.5% by MDS FISH | Donor | **AML** | *NRAS* p.Q61p (VAF = 32.9); DNMT3A p.R882C (VAF = 42.4); RUNX1 p.W106L (VAF = 31.8); RUNX1 p.A149fs (VAF = 29.6); TET2 p.R1134fs (VAF = 37.6) |
|  |  |  |  |  |  |  |
|  |  |  |  |  |  |  |
|  |  |  |  |  |  |  |
|  |  |  |  |  |  |  |
|  | +1521 |  | normal 7 | Donor | NEL | *DNMT3A* p.R882C (VAF = 11.9); TET2 p.R1134fs (VAF = 10.2) |
|  |  |  |  |  |  |  |
|  | +1596 | //46,X,der(Y)t(Y;1)(q11.23;q12)[9]//46,XY[11] | Normal 7 | Donor | NEL |  |
|  | +1745 | //46,X,der(Y)t(Y;1)(q11.23;q12)[18]//46,XY[2] | Normal 7 | Donor | Atypical megakaryo-cytes | *DNMT3A* p.R882C (VAF = 21.2); TET2 p.R1134fs (VAF = 22.6) |
|  |  |  |  |  |  |  |
|  | +1955 | ***Deceased*** | | | | |
| **D-2** | +28 |  |  | Donor | NEL |  |
|  | +94 | //46,XY[20] |  | Donor |  |  |
|  | +251 | //46,XY[20] |  | Donor |  |  |
|  | +2327 | //45,XY,-7[16]//46,XY[4] | -7 in 68.5% by MDS FISH | Donor | **MDS** | *CBL* p.C401Y (VAF= 7.1); ASXL1 p.G644fs (VAF = 25.5); SETBP1 p.T873K (VAF = 27.3);  IKZF1 p.V53M (VAF = 27.4); KMT2D p.R2370H (VAF = 49.3); PTPN11 p.T553M (VAF = 50.0) |
|  |  |  |  |  |  |  |
|  |  |  |  |  |  |  |
|  |  |  |  |  |  |  |
|  |  |  |  |  |  |  |
|  |  |  |  |  |  |  |
|  | +2537 | ***Deceased*** | | | | |
| **D-3** | +35, +64 |  |  | Donor | NEL |  |
|  | +76 | //46,XY[20] |  | Donor |  |  |
|  | +192 | //46,XY[20] |  | Donor | NEL |  |
|  | +372,+723, +993 |  |  | Donor |  |  |
|  | +1190 | //46,XY[20] | Normal MDS | Donor | NEL |  |
|  | +1842 |  |  | Donor |  |  |
|  | +3956 | //46,XY,+1,der(1;7)(q10;p10)[18]//46,XY[2] | 7q- in 38% | Donor | **AML** |  |
|  | +4166 | ***Deceased*** | | | | |
| **D-4** | +26 |  |  | Donor |  |  |
|  | +55 | //46,XY[20] |  | Donor |  |  |
|  | +192 | //46,XY[20] | Normal ALL FISH | Donor | NEL |  |
|  | +373 | //47~48,XY,+1~2mar[4]//46,XY[16] |  | Donor | **T-LGL** |  |
|  | +387 |  |  | Donor |  |  |
|  | +962 | ***Deceased*** | | | | |
| **D-5** | +28, +61 |  |  | Donor |  |  |
|  | +89 | //46,XX[11] |  | Donor | NEL | **BCL6* p.A517I (VAF = 44.7); **NSD1 p.A976V (VAF = 49.3) |
|  |  |  |  |  |  |  |
|  | +178 | //46,XX[20] |  | Donor | NEL |  |
|  | +426 | //46,XX,t(11;19)(q23;p13.3)[8]//46,XX[12] |  | Donor | **AML** | **BCL6* p.A517I (VAF = 45.0); **NSD1 p.A976V (VAF = 49.5) |
|  |  |  |  |  |  |  |
|  | +434 | //46,XX,t(11;19)(q23;p13.?3)[17]//46,XX[3] | *KMT2A* rearrange-ment in 14% | Donor | **AML** | **BCL6* p.A517I (VAF = 42.7); **NSD1 p.A976V (VAF = 46.6) |
|  |  |  |  |  |  |  |
|  | +473 | //46,XX,t(11;19)(q23;p13.3)[1]//46,XX[19] | Normal *KMT2A* | Donor | NEL | **BCL6* p.A517I (VAF = 43.2); **NSD1 p.A976V (VAF = 46.6) |
|  |  |  |  |  |  |  |
|  | +523 | //46,XX,t(11;19)(q23;p13.3)[9]//46,XX[11] |  | Donor | NEL |  |
|  | +602 |  | *KMT2A* rearrange-ment in 24% | Donor |  | **BCL6* p.A517I (VAF = 39.3); **NSD1 p.A976V (VAF = 51.3) |
|  | +676 | ***Deceased*** | | | |  |
| **D-6** | +28 | //46,XX[20] |  | Donor | NEL |  |
|  | +56, +116 |  |  | Donor |  |  |
|  | +154 | //46,XX[20] |  | Donor | NEL |  |
|  | +308, +483, +694, +1261, +1806, +2170, +2633 |  |  | Donor |  |  |
|  | +2680 | //46,XX,del(20)(q11.2q13.2)[7]//46,XX,der(21;22)(q10;q10)x2,+21,+22[11]  //46,XX[2] |  | Donor | **AML** | *RUNX1* p.k110fs (VAF = 23.1);  *MPL*, p.H353R (VAF = 46.1); *NF1*, p.A2806S (VAF = 49.1) |
|  | +2725 | //46,XX[14] | 20q- in 10% | Donor | NEL | *RUNX1* p.k110fs (VAF = 1.6);  *MPL* p.H353R (VAF = 49.2);  *NF1* p.A2806S (VAF = 50.1) |
| AML: Acute myeloid leukemia; DCL: Donor cell leukemia; MDS: Myelodysplastic syndrome; T-LGL: T-cell large granular lymphocytic leukemia; VAF: Variant allele frequency; *: in donor only; **: in donor and patient | | | | | | |

**S3: Acquired/somatic donor cell-derived genetic abnormalities in cases without evidence of leukemia**

| **Case ID** | **Time after transplant (days)** | **Cytogenomic data** | **FISH / RT-PCR** | **T-cell chimerism** | **Pathology findings** |
| --- | --- | --- | --- | --- | --- |
| **C-1** | +30 |  |  | Donor |  |
|  | +57 | //46,XX[20] |  | Donor | No evidence of MDS |
|  | +197 | //46,XX[20] |  | Donor | No evidence of MDS |
|  | +363 | //46,XX,del(20)(q12q13.3)[5]//46,XX[15] |  | Donor | Occasional atypical megakaryocytes |
|  | +501 |  |  | Donor |  |
|  | +548 |  |  |  | Non-cancer related death |
| **C-2** | +30 |  |  | Donor |  |
|  | +61 | //46,XY[20] |  | Donor | No leukemia |
|  | +191 | //46,XY[20] |  | Donor | No leukemia |
|  | +360 | //46,XY[20] |  | Donor | No leukemia |
|  | +399 | //46,XY,del(20)(q13.1)[4]//46,XY[16] | 20q- in 10% by MDS FISH | Donor | No leukemia |
|  | +735 | //46,XY,del(20)(q13.1)[6]//46,XY[14] | 20q- in 14.6% by MDS FISH | Donor | No leukemia |
|  | +741 | //46,XY,del(20)(q13.1)[3]//46,XY[17] | 20q- in 23.8% by MDS FISH | Donor | No leukemia |
|  | +781 | //46,XY,del(20)(q13.1)[10]//46,XY[10] | 20q- in 21.6% by MDS FISH | Donor | No leukemia |
|  | +1092 | //46,XY,del(20)(q13.1)[6]//46,XY[14] |  | Donor | No leukemia |
|  | +1463 | //46,XY,del(20)(q13.1)[3]//46,XY[17] |  | Donor | No leukemia |
|  | +1862 | //46,XY,del(20)(q13.1)[10]//46,XY[10] |  | Donor | No leukemia |
|  | +5110 |  |  |  | No leukemia |
| **C-3: The 1st trans-plant** | +31 | 46,XX[20] |  | Donor 1 |  |
|  | +55 | 46,XX,del(20)(q11.2)[4]/46,XX[16] |  | Donor 1 | Erythroid predominance and atypical megakaryocytes |
|  | +211 | 46,XX,del(20)(q11.2)[11]/46,XX[8] |  | Donor 1 |  |
|  | +302 | 46,XX,del(20)(q11.2)[8]/45,XX,-7[1]/45,XX,t(1;8)(q22;p?21),-7[2]/46,XX[9] |  | Mixed chimera patient 9% | No leukemia |
|  | +371 |  | 20q- in 16% and -7 in 18% by MDS FISH | Mixed chimera patient 18% |  |
|  | +431 | no BCR-ABL transcripts by RT-PCR | -7 in 40% by MDS FISH | Mixed chimera patient 23% |  |
|  | +476 |  |  | Mixed chimera patient 53% |  |
|  | +575 | 45,XX,der(1)t(1;8)(q11;p21),der(8)t(1;8)inv(1)(q11q25),-7[20] | -7 in 78.5% and 20q- in 2.4% FISH |  | Therapy-related MDS (relapse) |
|  | +627 | 46,XX,del(20)(q11.2)[3]/46,XX[17] | 20q- in 2.8% & normal 7 | Donor 1 | Atypical megakaryocytes |
|  | +659 | 46,XX,del(20)(q11.2)[9]/46,XX[11] |  | Donor 1 | Atypical megakaryocytes |
| **C-3: The 2nd transplant** | +50 | //46,XY[20] |  | Donor 2 | No leukemia |
|  | +173 | //46,XY,del(20)(q11.2)[1]/46,sl,del(7)(q22q32)[2]//46,XY[19] | 7q- in 3.2% by MDS FISH | Donor 2 | No leukemia |
|  | +230 | //46,XY[20] |  | Donor 2 | No leukemia |
|  | +376 | //46,XY,add(2)(q35),del(7)(q22q32),del(20)(q11.2)[2]//46,XY[18] |  | Donor 2 | Erythroid dysplasia |
|  | +524 | //46,XY,add(2)(q35),del(7)(q11.2q22),add(13)(q34),del(20)(q11.2)[7] //46,XY[13] | 20q- in 15% & normal 7 | Donor 2 | Myeloid predominance and dyserythropoiesis |
|  | +720 | //46,XY,del(20)(q11.2)[1]/46,sl,add(2)(q35),del(7)(q11.2q22),add(13)(q 34)[2]//46,XY[17] | Normal MDS FISH | Donor 2 | Dyserythropoiesis |
|  | +848 |  |  | Donor 2 | No leukemia |
|  | +1086 | //46,XY[20] | Normal MDS FISH | Donor 2 | No leukemia |
|  | +1457 | //46,XY[20] | Normal MDS FISH | Donor 2 | No leukemia |
|  | +1671 to +3639 |  |  | Donor 2 | No leukemia |
| **C-4** | +31 | //46,XY[20] |  | Donor | No leukemia |
|  | +55 | //47,XY,+i(9)(p10)[19]//46,XY[1] | +9 and 4 copies of *CDKN2A* in 97% by ALL FISH | Donor | No leukemia |
|  | +94 | //47,XY,+i(9)(p10)[17]//46,XY[3] | +9 and 4 copies of *CDKN2A* in 93% by ALL FISH | Donor | No leukemia |
|  | +181 | //47,XY,+i(9)(p10)[20] | +9 and 4 copies of *CDKN2A* in 84% by ALL FISH | Donor | No leukemia |
|  | +272 | //47,XY,+i(9)(p10)[20] | No *BCR::ABL* fusion by RT-PCR | Donor | No leukemia |
|  | +354 | //47,XY,+i(9)(p10)[20] | No *BCR::ABL* fusion by RT-PCR | Donor | No leukemia |
|  | +363 |  | No *BCR::ABL* fusion by RT-PCR | Donor |  |

ALL-FISH panel included probes for 9p21 (*CDKN2A*), 9cen (D9Z1), 9q34 (*ABL*), 22q11.2 (*BCR*), and 11q23 (*3'MLL, 5'MLL*); MDS- FISH panel included probes for 5p15.2 (D5S23, D5S721), 5q31 (*EGR1*), 7cen (D7Z1), 7q31 (D7S522), 8cen (D8Z2), 11q23 (*3'MLL, 5'MLL*), 20q12 (D20S108), and 20q13.12 (D20S150).
