# Supplementary figures and images for: Donor cell-derived genetic abnormalities after sex mismatched allogeneic cell transplantation: a unique challenge of donor cell leukemia
Source: Blood Cancer J. 2023 Nov 6;13(1):163. doi: 10.1038/s41408-023-00938-z (PMC10625970; doi:10.1038/s41408-023-00938-z)

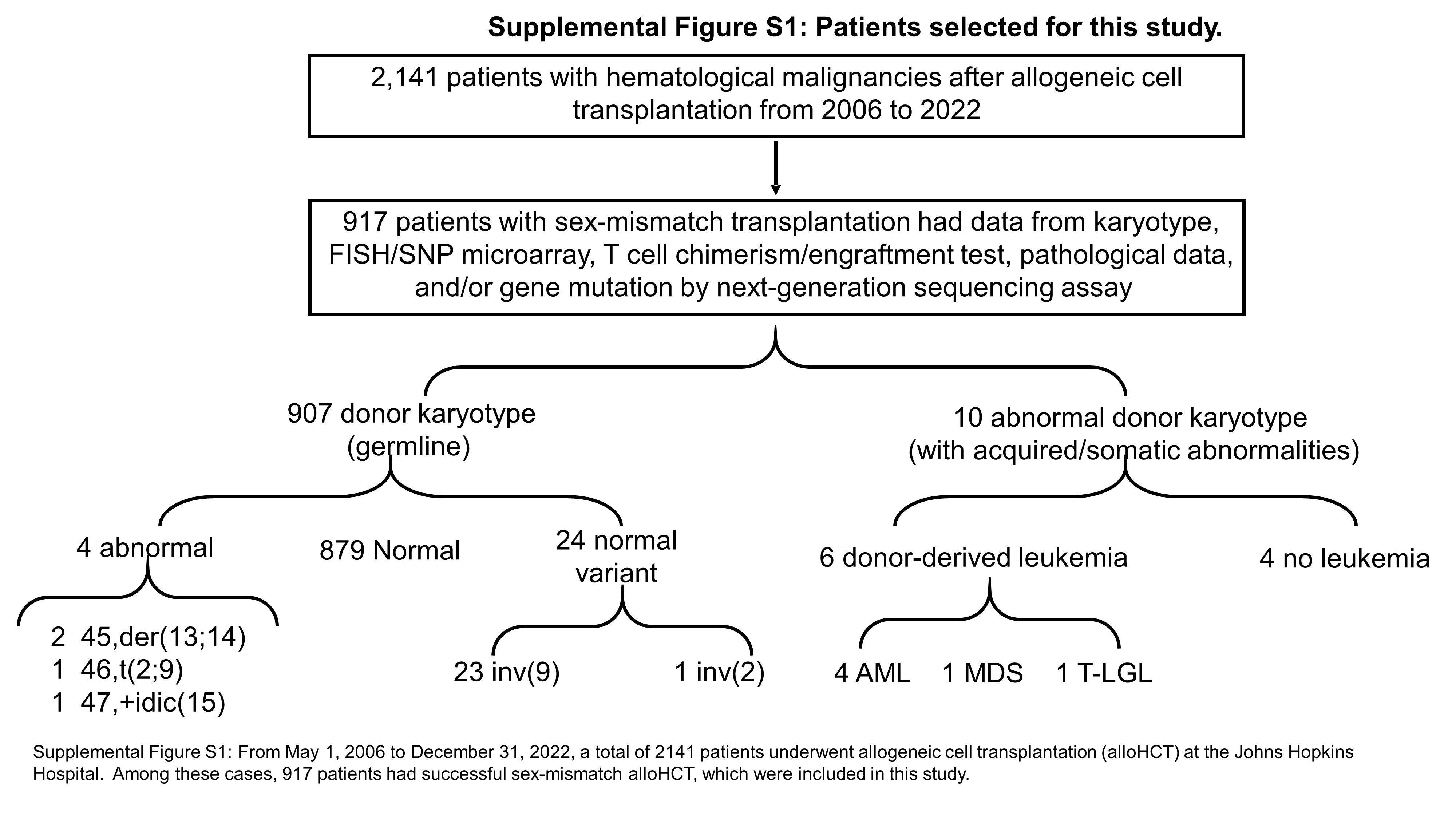

Supplement: Supplementary file 2 — Supplemental Figure S1 [file 41408_2023_938_MOESM2_ESM.jpg]

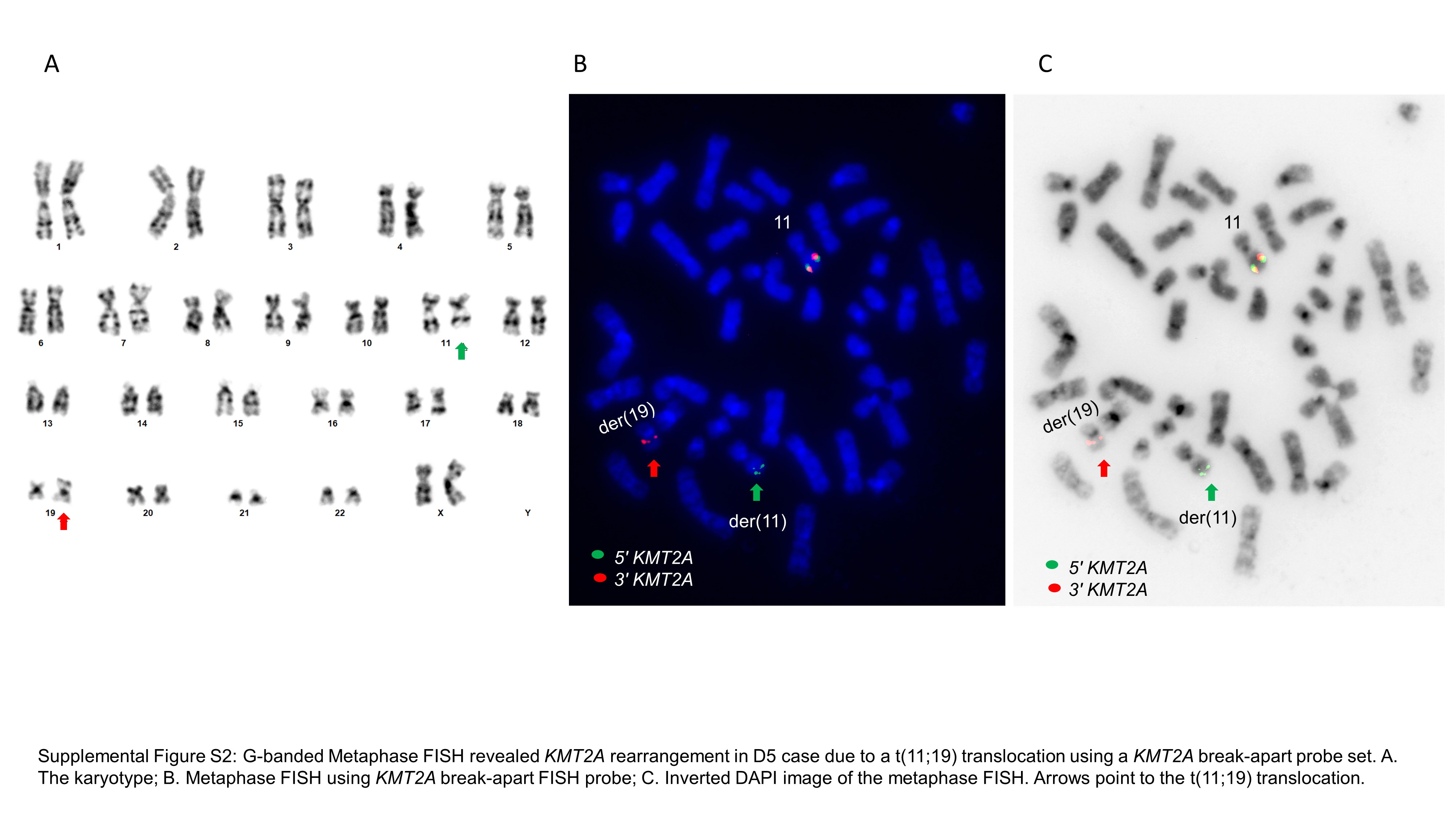

Supplement: Supplementary file 3 — Supplemental Figure S2 [file 41408_2023_938_MOESM3_ESM.jpg]
